# Individual Pig Identification Using Back Surface Point Clouds in 3D Vision

**DOI:** 10.3390/s23115156

**Published:** 2023-05-28

**Authors:** Hong Zhou, Qingda Li, Qiuju Xie

**Affiliations:** 1 College of Engineering, Heilongjiang Bayi Agricultural University, Daqing 163319, China; zh_hlj@126.com; 2 College of Electrical and Information, Northeast Agricultural University, Harbin 150030, China; 3 Key Laboratory of Swine Facilities Engineering, Ministry of Agriculture, Harbin 150030, China

**Keywords:** pig individual identification, 3D sensors, point clouds, PointNet++, deep learning

## Abstract

The individual identification of pigs is the basis for precision livestock farming (PLF), which can provide prerequisites for personalized feeding, disease monitoring, growth condition monitoring and behavior identification. Pig face recognition has the problem that pig face samples are difficult to collect and images are easily affected by the environment and body dirt. Due to this problem, we proposed a method for individual pig identification using three-dimension (3D) point clouds of the pig’s back surface. Firstly, a point cloud segmentation model based on the PointNet++ algorithm is established to segment the pig’s back point clouds from the complex background and use it as the input for individual recognition. Then, an individual pig recognition model based on the improved PointNet++LGG algorithm was constructed by increasing the adaptive global sampling radius, deepening the network structure and increasing the number of features to extract higher-dimensional features for accurate recognition of different individuals with similar body sizes. In total, 10,574 3D point cloud images of ten pigs were collected to construct the dataset. The experimental results showed that the accuracy of the individual pig identification model based on the PointNet++LGG algorithm reached 95.26%, which was 2.18%, 16.76% and 17.19% higher compared with the PointNet model, PointNet++SSG model and MSG model, respectively. Individual pig identification based on 3D point clouds of the back surface is effective. This approach is easy to integrate with functions such as body condition assessment and behavior recognition, and is conducive to the development of precision livestock farming.

## 1. Introduction

With the development of farm intensification, precision livestock farming (PLF) holds an increasingly important position in farm management [1]. The individual identification of different animals is a prerequisite for obtaining accurate body condition data of different individuals, which can enable precise control such as disease monitoring, precise feeding, and personalized management [2], and also help to improve animal welfare [3]. Individual identification is also the first step to achieve traceability technology in the animal product supply chain [4], and can provide a more accurate reference for animal insurance [5].

Traditionally, methods of individual identification of pigs mainly use radio frequency identification (RFID), and RFID [6] chips are implanted in ear tags or collars for individual animal identification. The contact identification causes pain and tends to induce stress in animals. In addition, the cost of additional labor is required for tag installation and separation, and tags can also be lost and worn out with long-term use [7]. With the development of sensors [8,9] and machine vision technology [10,11], it has become possible to apply non-contact machine vision methods of individual animal identification [12,13].

Individual pig identification based on machine vision includes manual marker recognition, facial recognition and body shape recognition. The early approach was to mark features such as numbers, colors and patterns on the back of pigs and then use pattern recognition techniques to distinguish different individuals [14]. However, this approach suffers from the problems of easy erasure of marker symbols and mutual occlusion between individuals [15].

In recent years, some studies have applied deep learning methods to pig face recognition by using the skin and texture of pig faces as features for individual pig recognition. Frontal pig face images were collected and a convolutional neural network (CNN) pig face recognition model with higher accuracy was established in comparison with Fisherfaces and VGG-Face algorithms [16]. A method for automatic screening of pig face images was explored [17], and a pig face recognition model for different growth stages was established [18]. To further improve the performance of pig face recognition models based on deep learning, most of the current research has focused on optimizing deep learning algorithms to improve the accuracy of pig face recognition and reducing the number of parameters of the models [19,20]. Facial recognition requires the cooperation of the object to be recognized. For example, human facial recognition usually requires the acquisition of a complete face image to obtain a high recognition accuracy [21]. However, due to the pigs’ natural tendency to move, it is difficult to look directly at the camera, and it is difficult to collect a standard frontal image of the pig face. At the same time, pig faces are often obscured by dirt, which poses a challenge for pig face recognition [17].

Some other studies have used the texture and color of the body skin and body size as the main features for individual pig identification [22,23]. An individual recognition model featuring the pig body was developed based on the YOLOv4 model used for target detection by collecting pictures of the pig body from multiple angles in a complex background [24]. A model for pig body segmentation and individual identification in different stacking states was developed [25]. In most of these studies, two-dimension (2D) RGB images were used as model inputs to extract body color, hair, texture and shape features for individual recognition. However, different from the rich markings of cows [26], the color of the whole body of pigs is relatively consistent, and they do not have obvious skin features [27]. The complexity of the background, the intensity of the light, the camera angle and alignment, and the soiling of the pig’s face and body surface can affect the accuracy of individual recognition during feature collection and are greater challenges for non-contact individual recognition techniques [28].

In recent years, with the development of three-dimension (3D) visual technology, image processing algorithms and computing hardware, the use of 3D images in PLF has been increasing [29,30]. Three-dimensional images are generally acquired by depth sensors or light detection and ranging (LiDAR) sensors, using binocular parallax, time-of-flight (TOF), and structured light for imaging [31]. Due to the inclusion of height information, 3D images can express more dimensional features than 2D images and are less affected by the environment and lighting [32]. Currently, 3D vision technology has a wide range of applications and good prospects in the industry [33], agriculture [34], and life fields such as face recognition [35], robotics [36], and automatic driving [37]. In PLF, 3D images are commonly used for 3D body surface reconstruction [38,39], monitoring of body conditions such as body size and weight [40], and also gradually applied for posture recognition [41], but few studies have been applied for individual identification.

Compared with pig face images, pig back surface images are more stable and easier to acquire. The point cloud of the pig’s back surface contains information about the width, length and height of different parts of the pig, which can represent the body size of the pig more comprehensively and is less affected by the environment, light and dirt on the pig’s body surface than 2D images. Therefore, we propose to establish an individual identification model based on the point clouds of pig back surface and automatically extract the 3D body shape features from the 3D point cloud data to provide a new idea for exploring the non-contact pig individual identification method.

## 2. Materials and Methods

### 2.1. Pigs and Housing

The 3D point cloud images of pigs used in this paper were collected from a free-range fattening barn in a pig farm in Wangkui County, Heilongjiang Province, China. The building style of the pig house is a semi-enclosed type with windows. Under natural ventilation and natural light conditions, 10 pigs were randomly selected for image and data acquisition. Different individuals were distinguished by marking symbols. Data were collected twice on 1 July and 10 July 2022, with the data collected on July 1 being training and validation data and the data collected on July 10 being test data. The breed of pigs is a crossbreed (Landrace × Large White), 110 d to 150 d old, weight range of 60 kg to 90 kg. The pigs are white in color with dirt on the body surface. The color of the floor and walls of the pig house is light gray.

### 2.2. Data Collection

A depth camera (model ORBBEC Astra Pro) was used to acquire color and depth images. The camera captures images by combining an infrared laser emitting source with an infrared-sensitive camera, using a binocular camera and utilizing the TOF principle. Both RGB and depth images were acquired at a resolution of 640 × 480 pixels with a frame rate of 30 fps. The horizontal and vertical fields of view are 66.1° and 40.2° for color images and 58.4° and 45.5° for depth images, respectively.

**Figure 1:**
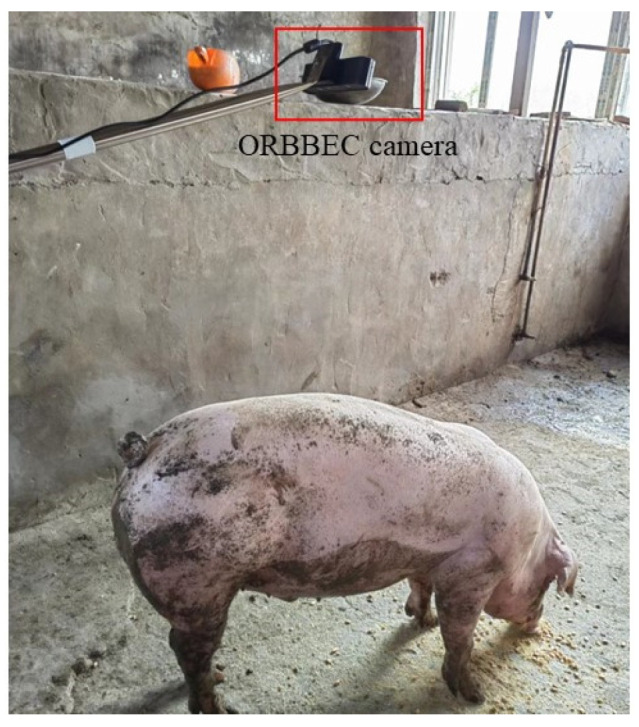
Location of image acquisition equipment.

For image acquisition, the depth camera is fixed on a retractable stand and shot vertically above the pig’s back to capture a top-down depth image of the pig with its back straight in a relatively stationary standing position. The location of the image acquisition equipment is shown in Figure 1. The camera is connected to the control computer via the USB port. Manual control of video capture using the computer. RGB and depth images were stored by frame. Weight was measured using a scale with a range of 0 to 800 kg and an accuracy of 0.5 kg. Body size was measured using a tape measure and measuring stick.

### 2.3. Dataset Description and Processing

The body weight (BW), chest width (CW), hip width (HW), chest height (CH), hip height (HH) and body length (BL) data of the 10 pigs are shown in Table 1. Among them, pig1, pig2, pig6 and pig7 are more similar in body size, pig3, pig4 and pig5 are more similar in body size, and pig8, pig9 and pig10 are more similar in body size.

Image filtering was performed manually by color images, and then the corresponding depth images were retained based on the filtered color images. To avoid the interference of color information such as light and pig surface dirt, only depth images were retained after screening, and color images were not used as data for this study. This study relied only on the features of body shape not on color features for individual identification. Image selection was based on the following criteria:

- The image contained the complete pig body;
- The posture of the pig was standing and the body was straight.

A point cloud image represents the shape of an object as a set of points, each point containing 3D position coordinates $\left(x,y,z\right)$. Since the depth image is two-dimensional in appearance and the point cloud image is three-dimensional in appearance, the point cloud image is more intuitive than the depth image. Standard depth images and point cloud images can be transformed to each other. In visual studio software (Version 2019; Microsoft Inc., USA), the OpenCV library and the PCL library were used to convert depth images to point clouds based on the intrinsic parameters of the camera. We used a checkerboard to calibrate the camera and obtain the focal length of the camera, $f_{x}=601.9267$, $f_{y}=603.3360$. The conversion of depth image to point cloud is to convert the data from image coordinate system to world coordinate system. The calculation formula is as follows:

(1) $$\left[\begin{array}{c} x\\ y\\ z \end{array}\right]=D\left[\begin{array}{ccc} \frac{1}{f_{x}} & 0 & 0\\ 0 & \frac{1}{f_{y}} & 0\\ 0 & 0 & 1 \end{array}\right]\left[\begin{array}{c} \;x^{\prime }\\ \;y^{\prime }\\ 1 \end{array}\right]$$

where x,y,z are the point cloud coordinates, D is the depth value, $f_{x}$ and $f_{y}$ are the camera focal lengths, and $\;x^{\prime }$ and $\;y^{\prime }$ are the depth image coordinates.

**Figure 2:**
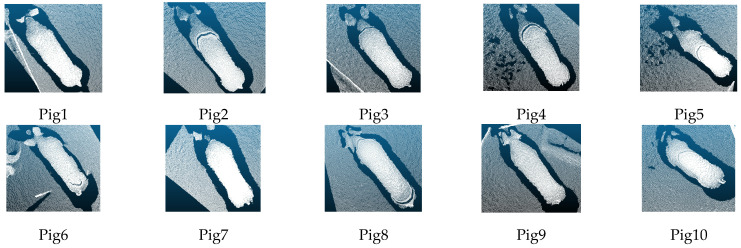
Top view of the point cloud of the pig’s back.

After converting the depth image into a point cloud image, the number of points of adjacent frames was determined automatically, and when the number of points of adjacent frames was the same, the frames were identified as duplicate frames and deleted automatically. The transformed point cloud contains the background and the pig body. The top view of the partial point cloud of 10 pigs containing the background is shown in Figure 2.

A total of 10,574 images were collected and filtered. A total of 8462 images collected and screened on 1 July were divided into a training set and a validation set in a ratio of 3:1. The 2112 images collected and screened on July 10 were used as the test set. The division of each dataset and the number distribution of point cloud images for each pig are shown in Table 2. As can be seen from the table, the number of samples was more evenly distributed among the pigs except for pig3. At the time of image acquisition, pig3 captured fewer images than the other pigs, resulting in a smaller number of final samples. In the analysis of experimental results, the final segmentation results and individual identification results of pig3 were discussed.

### 2.4. Segmentation and Identification Methods

#### 2.4.1. Overall Flow

**Figure 3:**
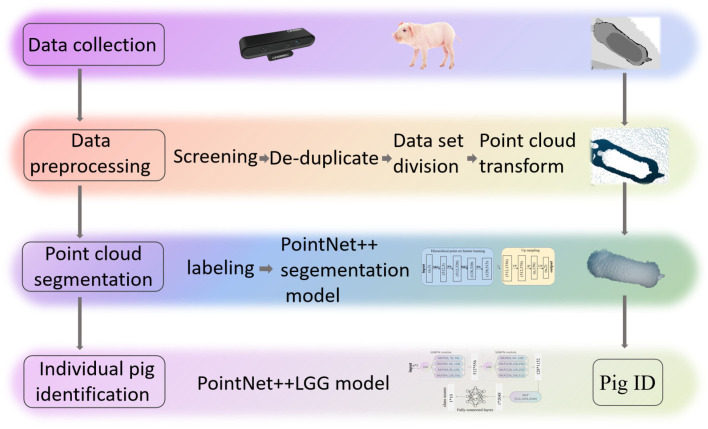
The process of pig individual identification based on the 3D point cloud of pig’s back surface.

Individual pig identification consists of two main parts: the construction of pig body segmentation model and the construction of individual identification model. Firstly, the acquired depth image is converted into a point cloud image after data pre-processing. Then, in order to reduce the interference of background and at the same time reduce the amount of input data for the recognition model, a PointNet++ point cloud segmentation model was built to segment the point cloud of the pig’s back. Finally, the point cloud on the back of the pig body was used as the input for individual identification through the Pointnet++LGG individual classification model, and the specific number of the pig was given. The process of pig individual identification based on the 3D point cloud of pig’s back surface is shown in Figure 3.

#### 2.4.2. Pig Body Segmentation Methods

The goal of pig body segmentation is to segment the pig back point cloud from the background point cloud. First, the back point cloud and background point cloud are labeled by software to obtain the true classification value of each data point. Then, a pig body segmentation model based on the PointNet++ algorithm was established to complete the segmentation of pig back point clouds.

1. Point cloud labeling

**Figure 4:**
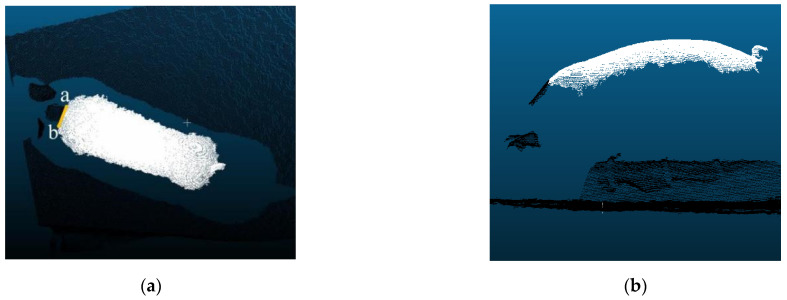
Pig point cloud segmentation markers, points a and b are head and neck segmentation points, 1 is marked as pig back and is shown in white, 0 is marked as background and is shown in black, (**a**) top view of the point cloud with segmented labels, and (**b**) side view of the point cloud with segmented labels.

The point clouds were labeled as the pig’s back point clouds and the background point clouds by using CloudCompare software. There were differences in the shape of pig heads collected from different individuals, and the point cloud images of some individual pig heads were incomplete. To avoid the interference of head features, the back body part of the pig with the head removed was used as the segmentation target. The pig head was identified by two characteristic points. The points with the greatest change in curvature at the junction of the neck and shoulder were used as the segmentation points. As shown in Figure 4, points a and b were head and neck division points. The two points were connected and the head of the pig was divided. Head and background were tagged as the same label. The infrared light from the camera could not penetrate the body of the pig, resulting in no point cloud data on the ground at the edge of the body. The blank area without point clouds in the top view of the back was used as a split edge. The point clouds in the vertical coverage area of the partition boundary line were marked as target point clouds. The vertical part contains almost no point cloud due to the occlusion of the pig’s body. As a result, the point clouds of the pig’s back were marked as a continuous whole.

2. Building a PointNet++ segmentation model

The PointNet [42] and PointNet++ [43] algorithms take points in the point clouds as input and extract features directly from the unstructured point clouds. The segmentation problem in this study is a part segmentation, and the target area of the segmentation is the back of the pig. The location of the point cloud is relatively concentrated, and there are no complex local features in other parts except for the detailed segmentation of the head and neck. Therefore, segmentation algorithms that pay excessive attention to local details are not needed. The PointNet and PointNet++ algorithms are classical point cloud algorithms that can meet the requirements of the segmentation problem in this study and are fast and concise. Compared with the PointNet algorithm, the PointNet++ algorithm takes local features into account and has better feature extraction capabilities. Therefore, the PointNet++ algorithm was chosen to build a pig body segmentation model in this study.

**Figure 5:**
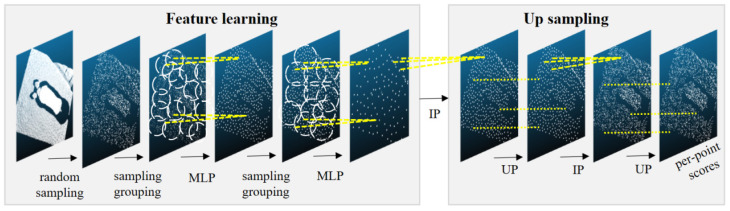
PointNet++ Segmentation Model. MLP: multilayer perceptron; IP: interpolation; UP: upsampling.

Since the number of point clouds in this study is very large, the original point clouds need to be sampled randomly, and the number of sampling points is n. The randomly sampled points are used as input to the model for feature extraction and segmentation. A set of 3D points $\left\{P_{i}|i=1,2,\ldots ,n\right\}$ is used as model input. Each point $P_{i}$ is a vector of $\left(x,y,z\right)$ coordinates without RGB information and normal vector information with input dimension n. The output is a classification label for each point, determining whether each point belongs to the pig body classification or the background classification. The output dimension is n ∗ 2. The pig body segmentation model established in this study is divided into two processes: downsampling for feature extraction and upsampling for feature propagation. Figure 5 shows the architecture of the segmentation model, with the feature extraction process on the left and the feature propagation process on the right.

In the process of feature extraction, n points are first randomly sampled from the input point cloud, and then two layers of sampling, grouping and feature extraction are performed. Among them, farthest point sampling is used as resampling method, single scale radius grouping is used as grouping method, and multilayer perceptron (MLP) is used to extract features to all points within the group. For a set of input points $\left\{P_{1},P_{2},\ldots ,P_{n}\right\}$, an ensemble function f is defined to represent the feature vector of the input point set. The feature extraction process is as follows:

(2) $$f(P_{1},P_{2},\ldots ,P_{n})=\gamma \left({\mathrm{MAX}}_{i=1,2,\ldots ,n}\left\{h\left(P_{i}\right)\right\}\right)$$

where $P_{i}$ is the input point, γ and h form the MLP network, and the max pooling function is used as the aggregation function.

In the process of feature propagation, a hierarchical propagation strategy with distance-based interpolation (IP) and cross-layer link hopping is used. After two IP and upsampling (UP) layers, the features are propagated to the original point set. Calculate the interpolation of each feature point based on the inverse distance weighted average of k-nearest neighbor. The features of the interpolation point x are noted as f. The calculation formula is as follows:

(3) $$f(x)=\frac{\sum_{i=1}^{k}\omega_{i}\left(x\right)f_{i}}{\sum_{i=1}^{k}\omega_{i}\left(x\right)}{\;\mathrm{where}\;\omega }_{i}\left(x\right)=\frac{1}{d{\left(x,x_{i}\right)}^{2}}$$

where $d\left(x,x_{i}\right)$ is the distance between the interpolation point x and the k nearest $x_{i}$ neighbors, $\omega_{i}\left(x\right)$ is the inverse distance.

#### 2.4.3. Individual Pig Recognition Methods

After completing the segmentation of the pig body, the segmented point clouds of pig back are used as input to identify different pig individuals by pig individual classification model. Using the basic single-scale grouping (SSG) strategy and multi-scale grouping (MSG) strategy, an individual recognition baseline model based on the PointNet++ classification algorithm is established. Since the problem in this study is classification between similar individuals of the same breed of pigs, an improved local-global grouping (LGG) strategy with a larger feature range and higher dimensions is proposed, and a PointNet++ LGG individual recognition model is established. From the segmented pig back point clouds, n points are randomly sampled and input to the model, and the input point clouds are represented as a set of 3D points $\left\{P_{i}|i=1,2,\ldots ,n\right\}$. Each point $P_{i}$ is a vector of $\left(x,y,z\right)\;$ coordinates. The dimensionality of the input data is n∗3. The output is the classification probability value of 10 pigs, so the output dimension is 1∗10. Compared with the segmentation model, the individual recognition model has only the process of downsampling and feature extraction, without the process of upsampling and feature propagation.

3. Individual pig identification model based on PointNet++ classification algorithm

**Figure 6:**
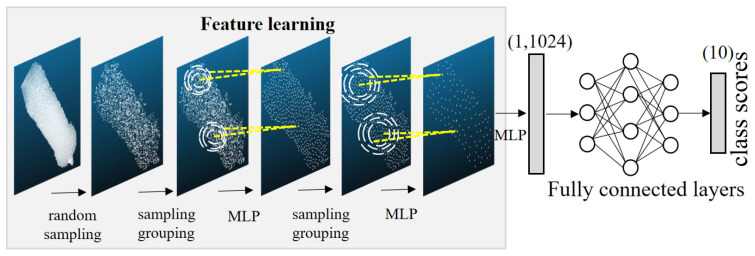
PointNet++ individual identification model. MLP: multilayer perceptron.

The structure of the individual recognition model of PointNet++ is shown in Figure 6. After two layers of sampling, grouping, and extracting features, the number of feature points is compressed from 2500 points to 128 points, and the feature dimension is raised from 3 to 643. Then the features are further compressed to 1024 features in one dimension by the MLP layer. After utilizing the fully connected layer and then the softmax function for classification, the problem of 10 classification is completed.

**Figure 7:**
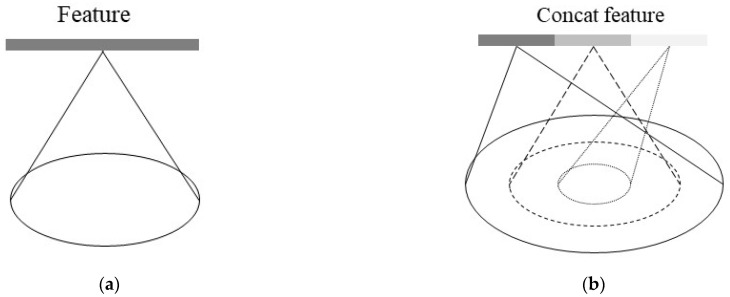
Principles of SSG and MSG, (**a**) SSG, and (**b**) MSG. SSG: single-scale grouping strategy; MSG: multi-scale grouping.

The SSG method uses a ball to query all points within a single sampling radius, and then the MLP is used to extract features from these points. However, due to the non-uniform density of random sampling points, feature extraction based on multi-scale radius plays an important role. The MSG sets different sampling radii $\left\{r_{1},r_{2},r_{3}\right\}$ to form multi-scale features by stringing together features of different scales. This sampling method helps to reduce the effect of sampling density. The principle of SSG and MSG is shown in Figure 7.

4. Improved pig individual recognition model based on the PointNet++ LGG classification algorithm

Since the pigs have more similar body size features, the classification model needs to learn to higher dimensional features to perform better differentiation between similar individuals. Since the pigs in this study were relatively similar in size, the classification model needed to learn higher dimensional features to better distinguish between similar individuals. In this paper, by using an improved local-global grouping strategy (LGG), we increase the range of feature extraction in PointNet++ grouping, enhance the dimensionality of feature extraction, and extract richer features of similar individuals.

**Figure 8:**
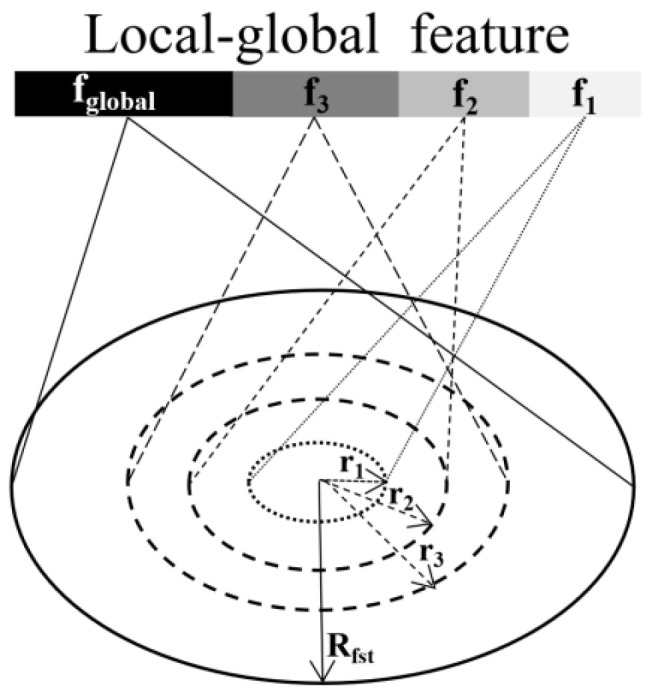
Improved LGG grouping strategy. LGG: local-global grouping strategy.

The principle of the improved grouping strategy LGG proposed in this paper is shown in Figure 8. Iterative farthest point sampling (FPS) is used to select point $\left\{x_{i_{1}},x_{i_{2}},\ldots ,x_{i_{m}}\right\}$ when the input point in the model sampling process is $\left\{x_{1},x_{2},\ldots ,x_{n}\right\}$ such that $x_{i_{j}}$ is the farthest point to the set $\left\{x_{i_{1}},x_{i_{2}},\ldots ,x_{i_{j}}-1\right\}$. The farthest distance between the sampled points is denoted as $R_{\mathrm{fst}}$. Make $R_{\mathrm{fst}}$ as the maximum sampling radius. When extracting the features, the global sampling radius $R_{\mathrm{fst}}$ is added to the local sampling radius $\left(r_{1},r_{2},r_{3}\right)$. Since the value of $R_{\mathrm{fst}}$ varies according to different samples, the feature extraction process adapts itself to different sampling samples.

In the improved LGG grouping strategy, the radius $\left\{r_{1},r_{2},r_{3},R_{\mathrm{fst}}\right\}$ is of different sizes and contains different numbers of sampling points, thus containing different amounts of information and extracting features of different dimensions. $\left\{f_{1},f_{2},f_{3},f_{\mathrm{global}}\right\}$ are the features at different sampling radii, and the dimensions are ordered from smallest to largest. MLP is used to extract features of different radii. Concatenate local features of different dimensions $\;\{f_{1},f_{2},f_{3}\}$ and global features $\left\{f_{\mathrm{global}}\right\}$. The feature of the point set consisting of m sampled points centered at point i is denoted as f($x_{i_{1}},x_{i_{2}},\ldots ,x_{i_{m}})$ and is calculated as follows:

(4) $$f(x_{i_{1}},x_{i_{2}},\ldots ,x_{i_{m}})=f_{j1}+f_{j2}+f_{j3}+f_{\mathrm{jglobal}},\;j=1,2,\ldots ,m$$

where $f_{j1}$, $f_{j2}$, $f_{j3}$ and $f_{\mathrm{jglobal}}$ are the characteristics of point j at radius $\left\{r_{1},r_{2},r_{3},R_{\mathrm{fst}}\right\}$, respectively.

The features $x_{i_{j}}$ of each point are extracted from its different neighborhood radii, and then the features under different radii are concatenated. The same method is used to extract features at different radii. $f_{\mathrm{jr}}$ denotes the characteristic of the point $x_{i_{j}}$ at radius r (r∈$\left\{r_{1},r_{2},r_{3},R_{\mathrm{fst}}\right\}$) and is calculated as follows:

(5) $$f_{\mathrm{jr}}=\gamma \left(h\left(∆p_{\mathrm{nj}}\right)\right),j\in N\left(j\right),r\in \left\{1,2,3,\mathrm{global}\right\}$$

where $N\left(j\right)$ is the neighbourhood point of point $x_{i_{j}}\;$ under radius r, $∆p_{\mathrm{nj}}$ is the relative coordinate of the neighbourhood point, h is the MLP process, and γ is the maximum polling function.

**Figure 9:**
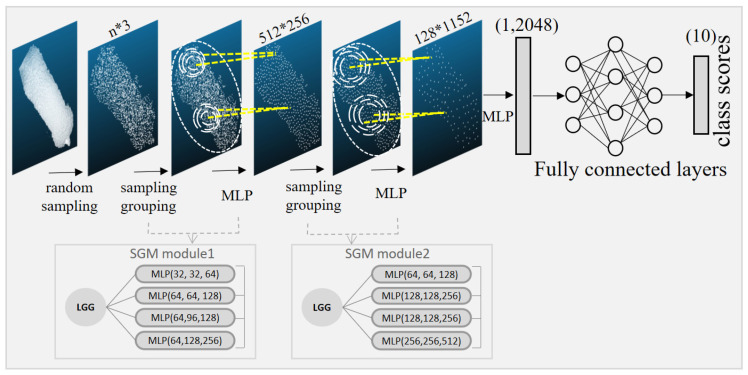
Improved pig individual recognition model based on PointNet++LGG. MLP: multilayer perceptron; SGM: sampling grouping module.

The structure of the PointNet++LGG improved pig individual recognition model is shown in Figure 9. The model uses the LGG grouping strategy with a total number of MLPs of 9. After two sampling grouping (SGM) and MLP feature extraction modules, the final generated feature dimension is 1 ∗ 2048. The increase in the number of model layers can extract higher and deeper classification features. The increase in feature dimension will better include these features. The final problem of 10 classification of similar individuals is completed by compressing the features through the fully connected layer and using the softmax classifier.

### 2.5. Experiment and Parameter Setting

The models were developed using Python 3.7.0 and libraries available in PyTorch 1.0.0. The computer was configured with 32 GB RAM, Windows 10 (64-bit), Intel i7-9700 3.0 GHz CPU, NVIDIA Tesla T4 GPU, and 16 GB discrete graphics memory. All models sampled 2500 points randomly on the original point cloud, normalizing them to a unit ball. Random rotation was used in the training to dynamically enhance the point cloud. The position of each point was dithered using Gaussian noise with a mean of zero and a standard deviation of 0.02.

In both the segmentation model and the individual recognition model, the Loss function adopts the Negative Log-Likelihood Loss (NLLLoss) function, the training learning rate is set to 0.001, the batch size is 8, the number of iterations is 50 and 150, respectively, and the models are optimized by Adam. In the PointNet++ individual recognition model, both SSG and MSG models adopt the default hyperparameters of the algorithm. The SSG model has two sampling radii of 0.2 and 0.4, and the number of sampling points of 32 and 64, respectively. The MSG model has sampling radii of [0.1, 0.2, 0.4] and [0.2, 0.4, 0.8], and the number of sampling points of [16, 32, 128] and [32, 64, 128], respectively. The sampling radii of LGG are [0.1, 0.2, 0.4, $R_{\mathrm{fst}}$] and [0.2, 0.4, 0.8, $R_{\mathrm{fst}}$], and the number of sampling points are [16, 32, 128, 256] and [32, 64, 128, 128], respectively, where $R_{\mathrm{fst}}$ is the variable, which is calculated based on the maximum distance of sampling points for different samples.

### 2.6. Evaluation Metrics

In the pig body segmentation model, overall segmentation accuracy (OA), mean intersection over union (mIoU), Precision, Recall, and F1 score were used to evaluate the performance of the segmentation model. OA was the ratio of correctly predicted points to the total number of points. Precision was the accuracy of pig body prediction. Recall was the proportion of pig body points that are correctly detected. F1 score was a combined evaluation index of Precision and Recall. IoU was the ratio of the predicted result to the true value. The IoU value of the pig body was defined as IoU_1_, and the IoU value of the background was defined as IoU_2_. mIoU was the average of the IoU of the pig body and the background.

False positive (FP) indicates pixels predicted to be pig body but with a false result. True positive (TP) indicates pixels predicted to be pig body with the true result. False negative (FN) indicates pixels predicted to be background but with a false result. True negative (TN) indicates pixels predicted to be a background with the true result. Accuracy, mIoU, Precision, Recall, and F1 score were defined as follows:

(6) $$\mathrm{Accuracy}=\frac{\mathrm{TP}+\mathrm{TN}}{\mathrm{TP}+\mathrm{TN}+\mathrm{FP}+\mathrm{FN}}$$

(7) $$\mathrm{Precision}=\frac{\mathrm{TP}}{\mathrm{TP}+\mathrm{FP}}$$

(8) $$\mathrm{Recall}=\frac{\mathrm{TP}}{\mathrm{TP}+\mathrm{FN}}$$

(9) $$F1\mathrm{score}=\frac{2\mathrm{Recall}*\mathrm{Precision}}{\mathrm{Recall}+\mathrm{Precision}}$$

(10) $${\mathrm{IoU}}_{i}=\frac{{\mathrm{TP}}_{i}}{{\mathrm{TP}}_{i}+{\mathrm{FP}}_{i}+{\mathrm{FN}}_{i}}$$

(11) $$\mathrm{mIoU}=\frac{1}{2}\left({\mathrm{IoU}}_{1}+{\mathrm{IoU}}_{2}\right)$$

In the individual recognition model, Accuracy, Precision, Recall, and F1 score were used to evaluate the performance of the pig individual recognition model as shown in Equations (6) to (9), respectively. TP was the number of correctly classified pigs in this category. FP was the number of incorrectly classified pigs in this category. TN was the number of correctly classified pigs that are not in this category. FN was the number of pigs that are not correctly classified that are not in this category.

## 3. Results and Discussion

### 3.1. Pig Body Segmentation

#### 3.1.1. Model Training Results

**Figure 10:**
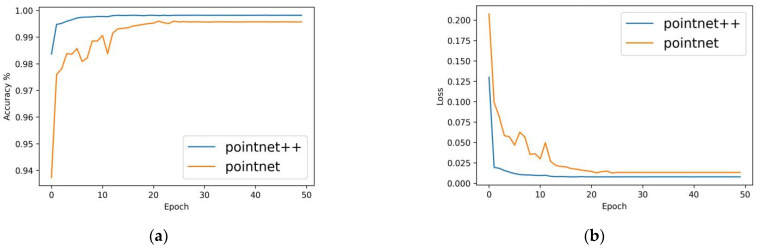
Accuracy and loss on validation set during training of the PointNet and PointNet++ segmentation models, (**a**) Accuracy, and (**b**) Loss.

In order to verify the performance of the PointNet++ pig body segmentation model established in this paper, a comparative analysis was made with the performance of the PointNet segmentation algorithm under the same data set and test environment. Figure 10 shows the test accuracy and loss value change curves of the validation set for different models during the training process. The PointNet++ segmentation model test accuracy ended up at around 99.81% with a loss value close to 0. The PointNet segmentation model ended up with an accuracy of around 99.56% and a loss value close to 0. It can be seen that both models have high accuracy, and both can complete the point cloud segmentation of pig body and background very well. The PointNet++ segmentation model was 0.25% more accurate than the PointNet segmentation model, and the loss value decreased more quickly.

#### 3.1.2. Model Test Results

The performance of the segmentation model on the test set was shown in Table 3. The OA of PointNet was 99.53% and that of PointNet++ was 99.80%. The overall segmentation accuracy of both models was high, where the OA of PointNet++ model was 0.27% higher than that of PointNet model. The mIoU, Precision, Recall and F1 scores of the PointNet++ model were slightly better than PointNet model with 0.81%, 1.25%, 0.25% and 0.67%, respectively. This did not indicate that the PointNet++ model showed better results than PointNet on every pig. There was a small randomness in the results of different pig segmentation, and this randomness generally originated from the random error when manually labeling the samples, and this error had a small random effect on the final segmentation effect.

**Figure 11:**
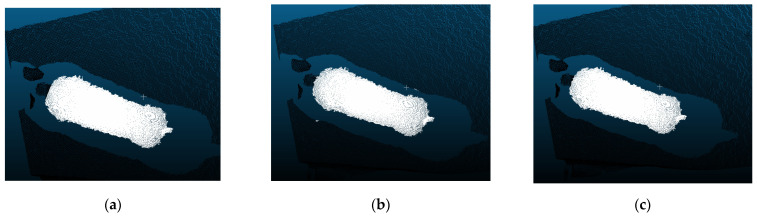
Segmentation effects for the PointNet model and PointNet++ model, (**a**) ground truth, (**b**) segmentation effect of the PointNet model, (**c**) segmentation effect of the PointNet++ model.

The segmentation effects of the PointNet and PointNet++ models were shown in Figure 11. Although there was a slight difference in their segmentation effect on the neck, it did not affect the overall profile and had a negligible effect on individual recognition. Both models successfully segmented the body and background of the pig.

In general, the PointNet++ model had good results in pig body point cloud segmentation, and demonstrated segmentation advantages in the head and neck segmentation part of the pig due to the algorithm itself focusing on local features. When the model was tested using separate datasets for each pig, there was no significant difference in segmentation between individuals, demonstrating that the PointNet++ model is robust to segmentation of the point clouds on the back of the pigs.

### 3.2. Individual Pig Identification

#### 3.2.1. Model Training Results

**Figure 12:**
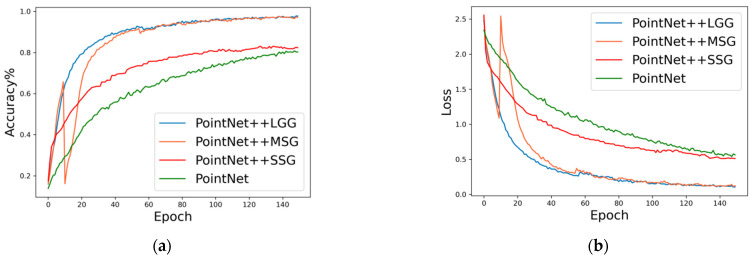
Accuracy and loss on validation set during training of the PointNet, PointNet++SSG, PointNet++MSG and PointNet++LGG identification models, (**a**) Accuracy, and (**b**) Loss.

As shown in Figure 12, the validation set accuracy grew during the training period of each model, and the loss values all decreased and finally converged. The PointNet model had an accuracy of 80% and a final loss value of 0.56. The PointNet++ SSG model had an accuracy of 82% and a final loss value of 0.51. The PointNet++ MSG model had an accuracy of 96 % and a final loss value of 0.12. The PointNet++ LGG model had an accuracy of 97% and a final loss value of 0.11.

It can be seen that the PointNet++LGG model proposed in this paper converged the fastest and had the highest accuracy, followed by the PointNet++MSG model, while the PointNet++ SSG model and the PointNet model had relatively low accuracy. Due to the large amount of point cloud data, each model used sampling to reduce the amount of data. The sampling method was random, which brought the problem of uneven point cloud distribution. Therefore, the feature extraction by multi-scale radius could be compatible with point cloud features of different densities and had better results in the experiment. The LGG model combined small-scale radius and global radius features, and set different feature dimensions to extract features according to the radius size, taking into account the feature extraction range and the amount of features, making the model able to obtain features of different individuals more quickly, so the PointNet++LGG model had the fastest convergence speed.

The final recognition accuracy of the model was affected by many factors. For example, sampling 2500 points from 50,000 to 60,000 points during the initial sampling inevitably resulted in some feature loss. Therefore, the PointNet++LGG model achieved a recognition accuracy of up to 97%, which had a high recognition performance.

#### 3.2.2. Model Test Results

The classification performance in the test set was shown in Table 4. The Accuracy, Precision, Recall and F1 scores of the PointNet++LGG model were 95.26%, 95.51%, 95.53% and 95.52%, respectively. They were higher than the PointNet++MSG model by 2.18%, 1.74%, 2.19%, 2.03%, the PointNet++SSG model by 16.76%,10.20%, 16.92%, 13.7%, and the PointNet model by 17.19%, 12.29%, 19.37%, 15.99%, respectively. The PointNet++ LGG model proposed in this paper had the best performance in classifying individual pigs at the overall level.

In order to evaluate the model performance from more scales and to understand the performance of each model in different categories, samples from each category were tested separately, and the results were shown in Table 5. The classification accuracy values of different pigs in the PointNet++LGG model, the highest being pig2 (100%) and the lowest being pig9 (85.64%), were similar to the overall classification accuracy (95.26%) and worked better for each classification dataset. The PointNet++MSG model had low classification accuracy for pig10 (78.33%). The PointNet++SSG model had low classification accuracy for pig10 (52.5%). The PointNet model had very low classification accuracy for pig1 (44.71%), which affected the final overall classification effect of the model. The low recognition rate of different models in a specific category might be caused by the similarity between samples from different categories. The uniformity of the recognition effect of PointNet++LGG model for different categories indicated that the model was compatible with different samples and had good stability.

**Figure 13:**
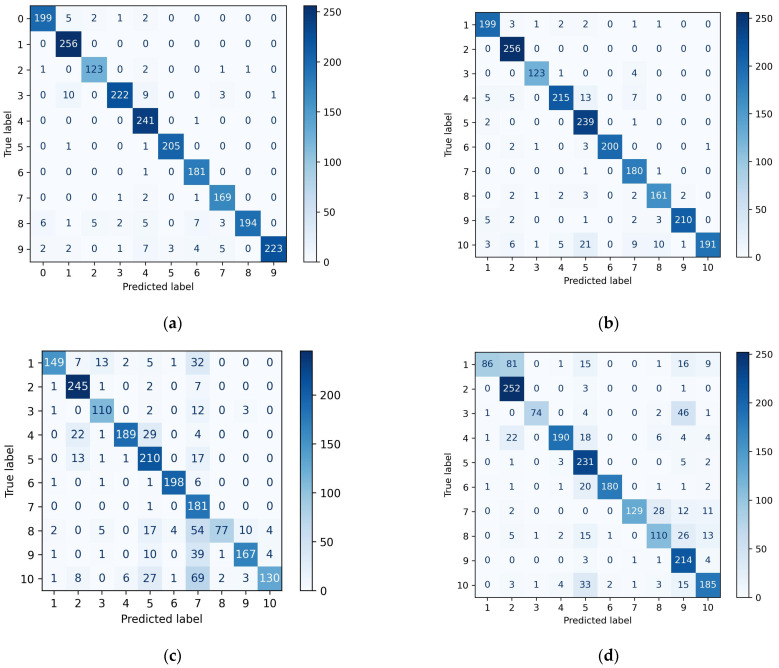
Confusion matrix for individual recognition models, (**a**) PointNet++LGG, (**b**) PointNet++MSG, (**c**) PointNet++SSG, and (**d**) PointNet.

In order to see more clearly the details of the classification results for each model for each pig, a classification confusion matrix was created and the results were shown in Figure 13. The predicted labels were represented by the horizontal coordinates of the matrix and the true labels were represented by the vertical coordinates of the matrix, with the values on the diagonal lines indicating the number of TPs, with higher values being darker in color. FN was the sum of the values in the horizontal coordinate with the diagonal position removed, FP was the sum of the values in the vertical coordinate with the diagonal position removed, and TN was the sum of the values in positions other than this row and column.

As shown in Figure 13a, the confusion matrix TP value of PointNet++LGG model was high, the recognition results were basically distributed on the diagonal, and the classification effect for each pig was relatively uniform, with a low probability of recognition error. As shown in Figure 13b, the values of the confusion matrix of the PointNet++MSG model were also mainly distributed on the diagonal. However, the FP value of pig5 was 44 relatively large, and other pigs were identified as pig5 more often. The FN value of pig10 was 56 relatively large, and the probability of being misidentified as other pigs was higher. As shown in Figure 13c, most of the values of the confusion matrix of the PointNet++SSG model were distributed on the diagonal. Pig1, pig8 and pig10 had larger FN values of 60, 96 and 117, respectively, and were more likely to be misidentified as other pigs. Pig7 had an FP value of 240, and other pigs were easily misidentified as this category. As shown in Figure 13d, in the confusion matrix of the PointNet model, FN values of pig1 and pig10 were large, 123 and 62, respectively. The FP values of pig2, pig5 and pig9 were larger, which were 115, 111 and 125, respectively.

To summarize the classification of different individuals, pig1 was more often identified as pig2, pig10 was more often identified as pig5 and pig7, and the number of other pigs identified as pig5 was more evenly distributed.

#### 3.2.3. Visual Analysis of Samples for Classification

**Figure 14:**
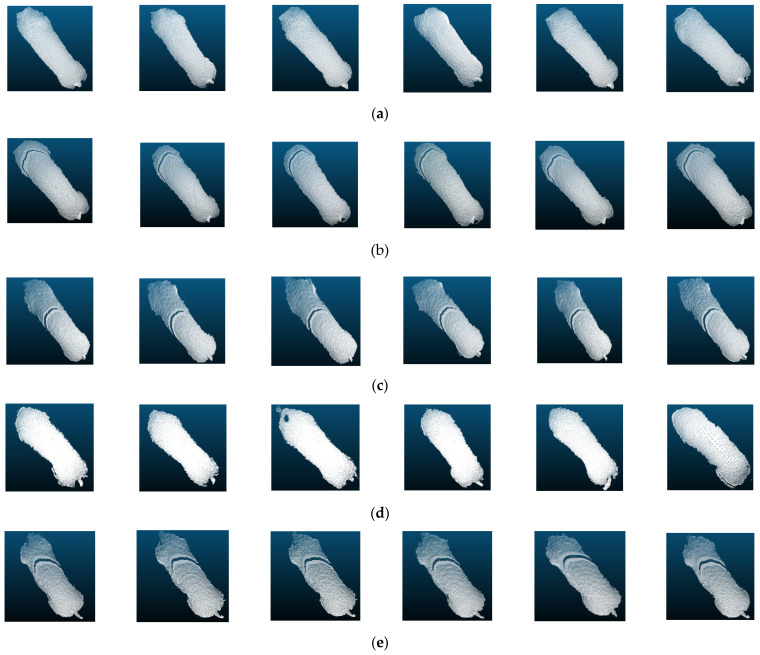
Visual analysis of samples for classification, (**a**) pig1, (**b**) pig2, (**c**) pig5, (**d**) pig7, and (**e**) pig10.

The similarity problem exhibited by the same individuals in different models might be related to the characteristics of the samples themselves. To explore the sources of variability in the recognition effects of different models in different categories, some samples in individual recognition were visualized. The top view of the segmented partial back point cloud image selected from pig1, pig2, pig5, pig7 and pig10 was shown in Figure 14. Combining the body size and weight data in Table 1, it could be seen that the body size and weight of pig1 and pig2 were similar and belonged to highly similar samples. The PointNet model often identified pig2 as pig1, indicating that the PointNet model has weak feature extraction ability for similar samples.

Pig10 differed significantly from pig5 and pig7 in body size and weight, and was misidentified probably because the model captured disturbing features other than body size. For example, the images of pig7 and pig10 were similar in the shape of the neck, and some images of pig5 and pig10 had cracks that were present on the body surface. These cracks were generated by infrared rays emitted by the depth camera at the greater curvature of the pig’s back and can generally be filled by pre-processing means such as point cloud complementation. In this paper, a deep learning algorithm was used to extract features automatically, and the segmented original point cloud was directly input into the model without any point cloud noise reduction process to achieve fully automatic recognition process. Therefore, the extraction of effective features and the filtering of invalid features was an important performance of individual recognition algorithms. From the experimental results, it could be seen that the PointNet model and the PointNet++SSG model easily identify pig10 as pig5 and pig7. The PointNet model only focused on the global information, and the PointNet++SSG model was less capable of learning the unevenly sampled point cloud features due to the use of a single feature extraction radius. Therefore, both models learned larger weights on the invalid features. The PointNet++MSG model, which incorrectly identified a small number of pig10 test samples as pig5, may also have focused too much on local information and assigned a certain weight on the invalid features.

The PointNet++LGG model proposed in this paper showed the highest classification accuracy compared with the other three models, and the classification effect for each pig was uniform, and the learning ability for similar samples was better, which indicated that the model had better stability.

### 3.3. Discussion

Unlike the point cloud segmentation of living scenes [44] and industrial scenes [33], the segmentation problem in this study was relatively simple. It was confirmed by experiment that the point cloud segmentation algorithm PointNet++ was used to successfully extract the point cloud of pig back with 99.8% accuracy, which had good segmentation effect. The segmentation of animal bodies by means of point cloud computation was performed in most previous studies. Wang et al. [45] collected point clouds of pig bodies for body size measurements and used a random sample consensus algorithm to remove ground point clouds. Shi et al. [38] collected 3D point clouds of the pig body for pig reconstruction, and the original point cloud consisted of target pigs, pens, ground and noise points. The railing and noise were removed by the point cloud filtering method, and then the ground was removed by the random sample consistency algorithm. The calculation process of this point cloud calculation is more complicated and requires different methods to remove the ground, walls and fences in the background. The PointNet++ pig body segmentation model constructed in this paper took the original point cloud collected as an input, and the model automatically completed the segmentation of the pig body, which was a very convenient process.

The dataset commonly used in classical point cloud classification algorithms such as the PointNet and PointNet++ algorithms was ModelNet40 [46]. The dataset used for the individual identification problem in this paper differed from its comparison in three ways. First, ModelNet40 distinguishes between different kinds of objects, while the objects in this study were different individuals of the same breed of pigs, and there was great similarity between the samples, which increased the difficulty of model classification. Second, the object in ModelNet40 had 10,000 points, while the segmented point cloud image of the pig’s back had 50,000–60,000 points, which added to the sampling challenge. Third, all samples in ModelNet40 were standard samples with uniform distribution. The samples in this study contain noise and cracks, which were not pre-processed and were used directly as input to the model to build a fully automated individual recognition system. This imposed higher requirements on the feature extraction ability and generalization ability of the model. The effect of different sampling grouping strategies on the classification effect of the model was compared experimentally. The results showed that the PointNet++LGG model constructed in this paper, which considered global features, achieved a recognition accuracy of 95.26%. PointNet++LGG classified all individuals uniformly, showing stronger feature extraction and better generalization ability. The addition of the LGG strategy in the PointNet++LGG model increased the feature extraction range of the model, deepened the structure of the network and increased the number of feature points, filtered out interfering features, and extracted higher-dimensional features used to distinguish similar individuals.

Individual identification has shown an important role in PLF and is the basis for achieving precision feeding and precision management. Coupling machine vision-based individual recognition with weight estimation, body size estimation, and behavior estimation in the same model, integrated in edge devices, will greatly improve the efficiency of farm management. Distinguishing individuals by pig face recognition, on the one hand, has the problem of difficult picture acquisition, and on the other hand, the analysis of other aspects in precision farming cannot be accomplished by pig face. At the same time, 3D data can provide richer features than 2D data, and now has a large number of applications in precision farming. Andrea Pezzuolo et al. [47] used a Kinect camera to measure body size based on pig back point cloud. Li et al. [48] calculated body size based on pig back point cloud data to build a regression model between body weight and body size. He et al. [49] built a body weight estimation model based on a pig back point cloud by a deep learning algorithm. Song et al. [31] calculated body size based on a cow back point cloud. The method of individual identification based on back 3D point cloud data proposed in this study belongs to a new exploration in the field of animal individual identification, which provides possible ideas for the integration of PLF functions in several aspects.

This study is a preliminary exploration of individual recognition using 3D body shape features and has some limitations. Experiments were conducted on individual recognition of ten pigs, and as the number of pigs increases, it will bring new challenges to the algorithm, and the number of pigs can be considered to increase in subsequent studies. In addition, how much the change in the appearance of the pigs affects the classification results over time is also a factor to be considered in subsequent experiments.

## 4. Conclusions

The following conclusions were drawn in this study:

1. A fully automated method of non-contact identification of individual pigs based on 3D point cloud of the pig back in two stages was proposed. The PointNet++ pig body segmentation model was established to segment the pig back point cloud from the background, and the segmentation accuracy reached 99.80%.
2. The PointNet++LGG algorithm with improved grouping strategy was proposed. The Accuracy, Precision, Recall and F1 score in individual recognition reached 95.26%, 95.51%, 95.53% and 95.52%, respectively. Compared with the PointNet algorithm, PointNet++SSG algorithm and PointNet++MSG algorithm, the recognition accuracy was higher, the recognition of similar individuals was better, the recognition of different individuals was more uniform, and the generalization ability was stronger.
3. The proposed method of pig individual recognition based on three-dimensional point cloud image of the back was a new exploration in the field of animal individual recognition, avoiding the stress reaction to the animal by radio frequency identification (RFID), avoiding the problem of difficult to obtain pig face samples in pig face recognition, and providing a new method and idea for individual recognition of other animals.

## Figures and Tables

**Table 1:** Weight and body size of 10 pigs. BW: body weight; CW: chest width; HW: hip width; CH: chest height; HH: hip height; BL: body length.

Body Size	Pig1	Pig2	Pig3	Pig4	Pig5	Pig6	Pig7	Pig8	Pig9	Pig10
BW (kg)	89.0	87.5	75.0	76.5	77.5	88.0	89.5	63.5	69.0	60.5
CW (cm)	30.6	32.1	30.3	27.7	29.6	31.5	30.56	25.9	27.6	25.9
HW (cm)	29.0	30.1	25.5	28.1	26.0	28.5	31.28	23.8	23.6	28.0
CH (cm)	67.5	66.4	56.6	57.5	57.8	63.6	60.9	53.2	54.4	52.1
HH (cm)	68.1	68.3	56.9	57.9	58.9	65.6	62.7	55.3	55.7	56.2
BL (cm)	97.7	95.4	82.8	86.8	86.6	96.7	90.6	85.9	89.2	77.4

**Table 2:** Dataset division.

Dataset	Pig1	Pig2	Pig3	Pig4	Pig5	Pig6	Pig7	Pig8	Pig9	Pig10	Total
Training set	631	779	384	735	727	621	544	520	668	741	6350
Validation set	209	256	128	245	242	207	182	173	223	247	2112
Test set	209	256	128	245	242	207	182	173	223	247	2112

**Table 3:** Test results of the PointNet and PointNet++ segmentation models. OA: overall segmentation accuracy; mIoU: mean intersection over union.

Models	Evaluation Metrics	Total	Pig1	Pig2	Pig3	Pig4	Pig5	Pig6	Pig7	Pig8	Pig9	Pig10
PointNet	OA (%)	99.53										
	Precision (%)	97.92	98.56	99.59	94.78	96.97	99.04	98.70	99.73	92.47	99.59	96.73
	Recall (%)	99.73	99.93	99.95	99.93	99.97	99.99	99.94	99.09	99.48	99.79	99.73
	F1 score (%)	98.82	99.24	99.77	97.29	98.45	99.51	9931	99.41	95.85	99.69	98.21
	mIoU (%)	98.55	99.03	99.70	96.88	98.11	99.41	99.17	99.12	95.25	99.64	97.89
PointNet++	OA (%)	99.80										
	Precision (%)	99.17	99.12	99.68	98.00	98.03	99.49	99.26	99.58	99.20	99.67	98.98
	Recall (%)	99.98	99.90	99.96	99.76	99.94	99.97	99.95	99.72	99.28	99.72	99.71
	F1 score (%)	99.49	99.51	99.82	98.87	98.97	99.73	99.60	99.65	99.24	99.70	99.34
	mIoU (%)	99.36	99.37	99.76	98.68	98.75	99.67	99.52	99.48	99.11	99.65	99.22

**Table 4:** Comparison of the overall classification accuracy of different models.

Models	Accuracy (%)	Precision (%)	Recall (%)	F1Score
PointNet	78.07	83.22	76.16	79.53
PointNet++SSG	78.50	85.31	78.61	81.82
PointNet++MSG	93.08	93.77	93.34	93.55
PointNet++LGG (Improved model)	95.26	95.51	95.53	95.52

**Table 5:** Test results of the PointNet and PointNet ++ classification models.

Models	Accuracy(%)									
	Pig1	Pig2	Pig3	Pig4	Pig5	Pig6	Pig7	Pig8	Pig9	Pig10
PointNet	44.71	93.75	96.09	90.41	97.08	87.50	83.52	83.33	88.42	85.00
PointNet++(SSG)	68.26	95.31	88.28	77.50	87.50	93.00	100.00	50.00	71.75	52.50
PointNet++(MSG)	96.63	100.00	98.33	97.00	92.12	97.50	99.43	92.85	92.59	78.33
PointNet++(LGG) (Improved model)	90.86	100.00	94.53	89.58	99.16	99.00	99.43	97.61	85.64	90.00

## Data Availability

Not applicable.
